# Role of Birds in Dispersal of Etiologic Agents of Tick-borne Zoonoses, Spain, 2009

**DOI:** 10.3201/eid1807.111777

**Published:** 2012-07

**Authors:** Ana M. Palomar, Paula Santibáñez, David Mazuelas, Lidia Roncero, Sonia Santibáñez, Aránzazu Portillo, José A. Oteo

**Affiliations:** Hospital San Pedro–Centro de Investigación Biomédica de La Rioja, Logroño, Spain (A.M. Palomar, P. Santibáñez, S. Santibáñez, A. Portillo, J.A. Oteo);; and Environment Resources Inc., Logroño (D. Mazuelas, L. Roncero)

**Keywords:** Anaplasma phagocytophilum, birds, ticks, Borrelia burgdorferi, Rickettsia spp., Borrelia turdi, Ixodes arboricola, Rickettsia sibirica, Rickettsia vini, Spain, Rickettsia, vector-borne infections, rickettsiae

## Abstract

We amplified gene sequences from *Anaplasma phagocytophilum*, *Borrelia garinii*, *B. valaisiana*, *B. turdi*, *Rickettsia monacensis*, *R. helvetica*, *R. sibirica sibirica*, and *Rickettsia* spp. (including *Candidatus* Rickettsia vini) in ticks removed from birds in Spain. The findings support the role of passerine birds as possible dispersers of these tick-borne pathogens.

Hard ticks are a major vector of infectious diseases in industrialized countries. Several tick-borne bacterial diseases, such as Lyme disease, Mediterranean spotted fever, and tick-borne lymphadenopathy (also called Dermacentor-borne necrosis erythema and lymphadenopathy), are endemic to Spain. Furthermore, a few cases of human anaplasmosis and *Rickettsia monacensis* infection in humans have been diagnosed in Spain (*1**–**3*).

Birds are the preferred host for some tick species. As carriers of infected ticks, birds could be responsible for the spread of tick-borne bacteria that cause human anaplasmosis, Lyme disease, rickettsioses, and other diseases (*4*). Multiple studies support the conclusion or propose the hypothesis that birds play a role as reservoirs of *Anaplasma phagocytophilum*, *Borrelia burgdorferi*, and *Rickettsia* spp (*4**–**6*). Because the Iberian Peninsula plays a major role in the migratory routes of birds, we aimed to determine the presence and prevalence of *A. phagocytophilum*, *B. burgdorferi* sensu lato, and *Rickettsia* spp. in ticks removed from birds captured in northern Spain.

## The Study

During April–October 2009, bird bandings were conducted in the protected area of Finca Ribavellosa in La Rioja, Spain (42°14′N, 2°54′W). Ticks were collected from birds and classified through taxonomic keys (*7*) and molecular methods (*8*). DNA was individually extracted by using 2 incubations of 20 minutes each with ammonium hydroxide (1 mL of 25% ammonia and 19 mL of Milli-Q water that had been autoclaved) at 100°C and 90°C.

DNA extracts were used as templates for PCRs targeting fragment genes for tick classification and for bacteria detection (Table 1). Two negative controls, 1 containing water instead of template DNA and the other with template DNA but without primers, and a positive control (a tick extract, *A. phagocytophilum*, *B. burgdorferi* sensu stricto, or *R. slovaca*) were included in all PCRs. Amplification products were sequenced, and nucleotide sequences were compared with those available in GenBank by using a BLAST search (www.ncbi.nlm.nih.gov/blast/Blast.cgi). Phylogenetic and molecular evolutionary analyses were conducted by using MEGA4 (*16* in Technical Appendix).

**Table 1:** PCR primer pairs used in study of the role of birds in dispersal of etiologic agents of tick-borne zoonoses, Spain, 2009*

Bacteria	Gene target	Primer name	Primer sequence, 5′ → 3′	Amplified fragment, bp	Annealing temp., °C	Ref.
*Anaplasma* spp.	16S rRNA, nested	ge3a	CACATGCAAGTCGAACGGATTATTC	932	55	(*9*)
		ge10r	TTCCGTTAAGAAGGAT CTAATCTCC			
		ge9f	AACGGATTATTCTTTATAGCTTGCT	546	55	(*9*)
		ge2	GGCAGTATTAAAAGCAGCTCCAGG			
	*msp*	msp3F	CCAGCGTTTAGCAAGATAAGAG	334	56	(*10*)
		msp3R	GCCCAGTAACAACATCATAAGC			
*Borrelia* spp.	*flaB,* nested†	Outer 1	AARGAATTGGCAGTTCAATC	497	52	(*11*)
		Outer 2	GCATTTTCWATTTTAGCAAGTGATG			
		Inner 1	ACATATTCAGATGCAGACAGAGGTTCTA	389	55	(*11*)
		Inner 2	GAAGGTGCTGTAGCAGGTGCTGGCTGT			
	5S-23S intergenic spacer, nested	23SC1	TAAGCTGACTAATACTAATTACCC	380	52	(*12*)
		23SN1	ACCATAGACTCTTATTACTTTGAC			
		5SCB	GAGAGTAGGTTATTGCCAGGG	226	55	(*12*)
		23SN2	ACCATAGACTCTTATTACTTTGACCA			
*Rickettsia* spp.	*ompA,* seminested	Rr190.70p	ATGGCGAATATTTCTCCAAAA	631	46	(*13**,**14*)
		Rr190.701n	GTTCCGTTAATGGCAGCATCT			
		Rr190.70p	ATGGCGAATATTTCTCCAAAA	532	48	(*14*)
		Rr190.602n	AGTGCAGCATTCGCTCCCCCT			
	*ompB*, nested	rompB OF	GTAACCGGAAGTAATCGTTTCGTAA	511	54	(*15*)
		rompB OR	GCTTTATAACCAGCTAAACCACC			
		rompB SFG IF	GTTTAATACGTGCTGCTAACCAA	420	56	(*15*)
		rompB SFG/TG IR	GGTTTGGCCCATATACCATAAG			
	*gltA* central region, nested	RpCS.877p	GGGGGCCTGCTCACGGCGG	381	48	(*14*)
		RpCS1258n	ATTGCAAAAAGTACAGTGAACA			
		RpCS.896p	GGCTAATGAAGCAGTGATAA	337	54	(*15*)
		RpCS.1233n	GCGACGGTATACCCATAGC			

*Temp., temperature; ref., reference; *msp*, p44 major surface protein gene; *flaB*, flagellin gene; *ompB*, 120-kDa genus common antigen gene; *ompA*, 190-kDa protein antigen gene; *gltA*, citrate synthase gene. †R = A/G; W = A/T.

A total of 222 ticks belonging to the species *Haemaphysalis punctata* (n = 1), *Ixodes frontalis* (n = 7), *I. arboricola* (n = 26), *I. ricinus* (n = 181), and other *Ixodes* spp. (n = 7) were collected from 97 passerine birds. Two nucleotide sequences for the 16S rRNA fragment gene of *I. arboricola* ticks were recorded (GenBank accession nos. JF791812 and JF791813) (Table 2).

**Table 2:** *Anaplasma phagocytophilum*, *Borrelia burgdorferi* s.l., and *Rickettsia* spp. detected in ticks removed from birds, Spain, 2009

Bacteria	Tick		Bird species (no. specimens)	Gene targets
	Species	Stage		
*A. phagocytophilum*	*Ixodes ricinus*	1 L	*Turdus merula* (1)	*msp*
*B. garinii*	*I. ricinus*	4 L, 2 N	*T. merula* (9)	*flaB*, 5–23S is
		3 L, 4 N		*flaB* or 5–23S is
		1 L	*Erithacus rubecula* (1)	*flaB*
		1 L	*T. philomelos* (1)	*flaB*, 5–23S is
		1 L	*Troglodytes troglodytes* (1)	*flaB*, 5–23S is
	*I. frontalis*	1 F	*T. philomelos* (1)	*flaB*, 5–23S is
	*Ixodes* spp.	1 L	*E. rubecula* (1)	5–23S is
	*Haemaphysalis punctata*	1 L	*T. merula* (1)	*flaB*, 5–23S is
*B. valaisiana*	*Ixodes* spp.	1 L	*T. merula* (1)	*flaB*, 5–23S is
	*I. ricinus*	1 L, 1 N	*T. merula* (3)	*flaB*, 5–23S is
		2 L		*flaB*
		1 L, 1 N	*T. philomelos* (2)	*flaB*, 5–23S is
		1 L	*E. rubecula* (1)	*flaB*, 5–23S is
		1 L	*Garrulus glandarius* (1)	*flaB*
*B. turdi*	*I. frontalis*	1 F	*T. merula (*1*)*	*flaB*, 5–23S is
*R. monacensis*	*I. ricinus*	1 N	*Sylvia* *atricapilla* (1)	*ompA*
*R. helvetica*	*I. ricinus*	1 N	*G. glandarius* (1)	*gltA*
*R. sibirica sibirica*	*I. ricinus*	1 L	*S. atricapilla* (1)	*ompA*
*Rickettsia* spp.†	*I. ricinus*	1 N, 1 L	*T. philomelos* (1)	*ompB* or *gltA*
	*I. ricinus*	4 L	*E. rubecula* (4)	*ompB* or *gltA*
		2 N	*T. merula* (2)	*gltA*
		1 L	*Tr. troglodytes* (1)	*gltA*
*Candidatus* Rickettsia vini	*I. abroricola*	20 N	*Cyanistes caeruleus* (1)	*ompA*, *ompB*, *gltA*
		5 L	*Parus major* (1)	*ompA*, *ompB*, *gltA*
	*I. ricinus*	2 L	*E. rubecula* (2)	*ompA*, *ompB*, *gltA*

*L, larva; *msp*, p44 major surface protein gene; N, nymph; *flaB*, flagellin gene; 5S-23S is, 5S-23S rRNA intergenic spacer; *ompB*, 120-kDa genus common antigen gene; *ompA*, 190-kDa protein antigen gene; *gltA*, citrate synthase gene. †Same identity with >1 validly published *Rickettsia* species.

*A. phagocytophilum* was detected only in 1 larva of an *I. ricinus* tick (0.5%). Twenty-nine (13.1%) samples tested positive for *B. burgdorferi* s.l. The most prevalent genospecies was *B. garinii* (n = 19), which was detected in *I. ricinus* (n = 16), *H. punctata* (n = 1), *I. frontalis* (n = 1), and *Ixodes* sp. (n = 1) ticks. *B. valaisiana* was amplified in 9 samples (8 *I. ricinus* and 1 *Ixodes* sp. ticks). *B. turdi* was found in 1 *I. frontalis* tick. *Rickettsia* infection was detected in 39 (17.6%) ticks. *R. monacensis* (n = 1), *R. helvetica* (n = 1), *R. sibirica sibirica* (n = 1), and *Rickettsia* spp. (n = 9) were detected in 12 *I. ricinus* ticks. Furthermore, according to *gltA*, *ompA*, and *ompB* sequence analysis, a possible new *Rickettsia* sp. was found in 25 *I. arboricola* ticks and 2 *I. ricinus* ticks. For these 27 samples, highest identities with *R. heilongjiangensis* (97.1%) and *R. japonica* (99.1%) were found for *ompA* (GenBank accession no. JF758828) and *ompB* (GenBank accession no. JF758826) nucleotide sequences, respectively, whereas *gltA* nucleotide sequences were identical to those from both *Rickettsia* spp. According to multilocus sequence typing (data not shown) and genetic criteria agreed on by experts, a *Candidatus* status could be assigned. We named it *Candidatus* Rickettsia vini (*17* in Technical Appendix) (Table 2). The phylogenetic tree based on *ompA* gene shows the nearest relationships among *Rickettsia* spp. (Figure).

**Figure:**
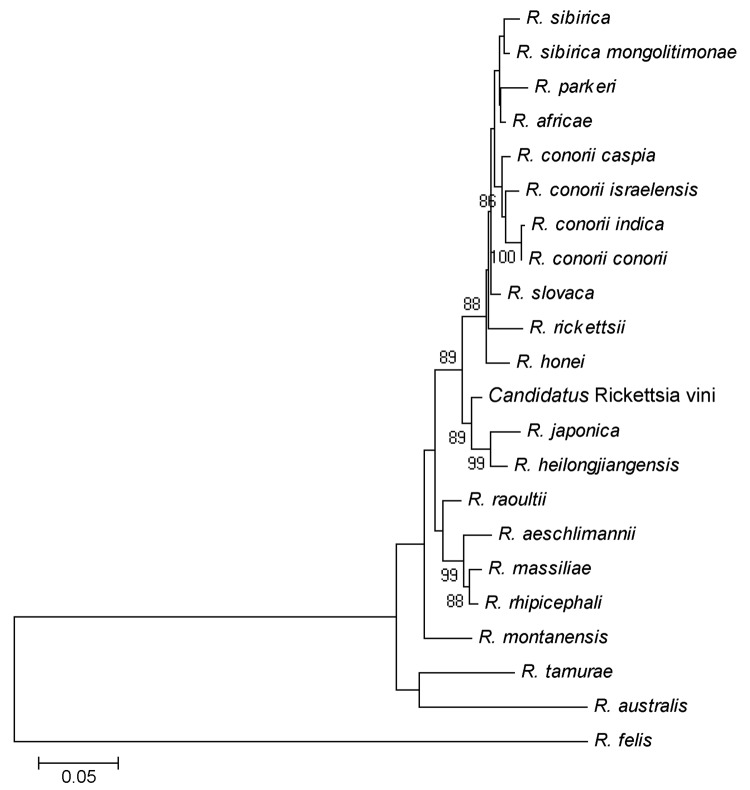
The phylogenetic position of *Candidatus* Rickettsia vini based on the *ompA* nucleotide sequences in a study of the role of birds in dispersal of etiologic agents of tick-borne zoonoses, Spain, 2009. The evolutionary history was inferred by using the neighbor-joining method. The optimal tree with the sum of branch length = 1.09961140 is shown. The percentage of replicate trees in which the associated taxa clustered in the bootstrap test (1,000 replicates) is shown next to the branches. The tree is drawn to scale, with branch lengths in the same units as those of the evolutionary distances used to infer the phylogenetic tree. The evolutionary distances were computed by using the Kimura 2-parameter method and are in the units of the number of base substitutions per site. Codon positions included were 1st+2nd+3rd+Noncoding. All positions containing gaps and missing data were eliminated from the dataset. A total of 563 positions were in the final dataset. Phylogenetic analyses were conducted in MEGA4 (*16* in Technical Appendix).

Two *I. ricinus* larvae showed co-infection with *B. garinii* and *Rickettsia* sp. One nymph was co-infected with *B. valaisiana* and *Rickettsia* sp.

## Conclusions

The presence of *Anaplasma*, *Borrelia*, and *Rickettsia* species in ticks removed from passerine birds corroborates the role of these vertebrates in the epidemiology and dispersion of tick-borne pathogens in Spain and in other zones of the planet. Some of the parasitized birds in our study, such as the European robin (*Erithacus rubecula*) or Eurasian blackcap (*Sylvia atricapilla*), are considered migratory or partial migratory birds. In addition, these species share an ecologic niche and ectoparasites (horizontal transmission) with other migratory birds that cover long distances from Africa to the Eurasian region.

Except for *I. arboricola*, the tick species captured in this study previously had been found on birds in Spain (*18* in Technical Appendix). Nevertheless, *I. arboricola* ticks are commonly hosted by birds. The high prevalence of *I. ricinus* ticks was expected because it is the most frequent tick in this area, and the immature stages of this tick frequently parasitize birds.

*I. ricinus* ticks are the main vectors of *A. phagocytophilum* in Europe, and this microorganism has been detected on vegetation in the studied area (*1*). However, the low prevalence (0.5%) of *A*. *phagocytophilum* in the ticks in our study corroborates data from other studies (*19*,*20* in Technical Appendix). The presence of *A. phagocytophilum* in a larva in our study supports the role of birds as reservoirs of *A. phagocytophilum*.

The prevalence (13.1%) of *B. burgdorferi* in our samples is similar to prevalences reported in other studies in Europe in which *I. ricinus* is the main species of tick captured from birds (*19* in Technical Appendix). In Spain, *B. garinii*, *B. valaisiana*, and *B. afzelii* have been detected in ticks from birds (*18* in Technical Appendix). According to our data, the human pathogen *B. garinii* was the most prevalent species, as reported in birds from Europe (*21* in Technical Appendix). *B. turdi* was discovered in Asia. Although it has been recently detected in ticks from birds in Norway (*22* in Technical Appendix), its finding in Spain was unexpected.

Regarding *Rickettsia* species, *R. monacensis* and *R. helvetica* are among the human pathogens detected in our study. Both species have been identified in ticks from birds in Europe (*19*,*20*,*23* in Technical Appendix). On the contrary, *Candidatus* Rickettsia vini, a potential new *Rickettsia* species, also detected in our study, has not been related to human disease (*17* in Technical Appendix). Several genospecies closely related to *R. heilongjiangensis* and *R. japonica* have been identified in *Ixodes* spp. ticks removed from birds (*23* in Technical Appendix). *R. sibirica sibirica*, responsible for Siberian tick typhus in western People’s Republic of China and in Siberia, was also amplified in an *I. ricinus* larva in this study.

Our data confirm the involvement of birds in the cycle of human tick-borne diseases. The findings confirm that birds can disperse vectors and microorganisms.

## Supplementary Material

Technical Appendix Additional references.
