# Supplementary material for: Role of Birds in Dispersal of Etiologic Agents of Tick-borne Zoonoses, Spain, 2009
Source: Emerg Infect Dis. 2012 Jul;18(7):1188–91. doi: 10.3201/eid1807.111777 (PMC3376802; doi:10.3201/eid1807.111777)
Supplement: Technical Appendix — Additional references. [file 11-1777-Techapp_2p.pdf]

Article DOI: http://dx.doi.org/10.3201/eid1807.111777

Role of Birds in Dispersal of Etiologic Agents of Tick-borne Zoonoses, Spain, 2009

Technical Appendix

References

16. Tamura K, Dudley J, Nei M, Kumar S. MEGA4: Molecular Evolutionary Genetics Analysis (MEGA) software version 4.0. Mol Biol Evol. 2007;24:1596–9. PubMed http://dx.doi.org/10.1093/molbev/msm092

17. Palomar AM, Portillo A, Santibáñez P, Santibáñez S, García-Álvarez L, Oteo JA. Genetic characterization of *Candidatus* Rickettsia vini. A new rickettsia amplified in ticks from La Rioja, Spain. Ticks Tick Borne Dis. 2012. In press.

18. Estrada-Peña A, Osácar JJ, Pichon B, Gray JS. Hosts and pathogen detection for immature stages of *Ixodes ricinus* (Acari: Ixodidae) in north-central Spain. Exp Appl Acarol. 2005;37:257–68. PubMed http://dx.doi.org/10.1007/s10493-005-3271-6

19. Franke J, Fritzsch J, Tomaso H, Straube E, Dorn W, Hildebrandt A. Coexistence of pathogens in host-seeking and feeding ticks within a single natural habitat in central Germany. Appl Environ Microbiol. 2010;76:6829–36. PubMed http://dx.doi.org/10.1128/AEM.01630-10

20. Santos-Silva MM, Sousa R, Santos AS, Melo P, Encarnação V, Bacellar F. Ticks parasitizing wild birds in Portugal: detection of *Rickettsia aeschlimannii*, *R. helvetica* and *R. massiliae*. Exp Appl Acarol. 2006;39:331–8. PubMed http://dx.doi.org/10.1007/s10493-006-9008-3

21. Comstedt P, Bergström S, Olsen B, Garpmo U, Marjavaara L, Mejlon H, et al. Migratory passerine birds as reservoirs of Lyme borreliosis in Europe. Emerg Infect Dis. 2006;12:1087–95. PubMed http://dx.doi.org/10.3201/eid1207.060127

22. Hasle G, Bjune GA, Midthjell L, Røed KH, Leinaas HP. Transport of *Ixodes ricinus* infected with *Borrelia* species to Norway by northward-migrating passerine birds. Ticks Tick Borne Dis. 2011;2:37–43. PubMed http://dx.doi.org/10.1016/j.ttbdis.2010.10.004

23. Elfving K, Olsen B, Bergström S, Waldenström J, Lundkvist A, Sjöstedt A, et al. Dissemination of spotted fever rickettsia agents in Europe by migrating birds. PLoS ONE. 2010;5:e8572. PubMed http://dx.doi.org/10.1371/journal.pone.0008572
